# Machine learning-driven clinical decision support system for concept-based searching: a field trial in a Norwegian hospital

**DOI:** 10.1186/s12911-023-02101-x

**Published:** 2023-01-10

**Authors:** G. T. Berge, O. C. Granmo, T. O. Tveit, B. E. Munkvold, A. L. Ruthjersen, J. Sharma

**Affiliations:** 1 Department of Information Systems, University of Agder, Kristiansand, Norway; 2 Department of Technology and eHealth, Sørlandet Hospital Trust, Kristiansand, Norway; 3 Department of ICT, University of Agder, Grimstad, Norway; 4 Department of Anaesthesia and Intensive Care, Sørlandet Hospital Trust, Kristiansand, Norway; 5 Research Department, Sørlandet Hospital Trust, Kristiansand, Norway

**Keywords:** Clinical decision support systems, Natural language processing, Technology acceptance, UTAUT, Machine learning, Electronic health record

## Abstract

**Background:**

Natural language processing (NLP) based clinical decision support systems (CDSSs) have demonstrated the ability to extract vital information from patient electronic health records (EHRs) to facilitate important decision support tasks. While obtaining accurate, medical domain interpretable results is crucial, it is demanding because real-world EHRs contain many inconsistencies and inaccuracies. Further, testing of such machine learning-based systems in clinical practice has received limited attention and are yet to be accepted by clinicians for regular use.

**Methods:**

We present our results from the evaluation of an NLP-driven CDSS developed and implemented in a Norwegian Hospital. The system incorporates unsupervised and supervised machine learning combined with rule-based algorithms for clinical concept-based searching to identify and classify allergies of concern for anesthesia and intensive care. The system also implements a semi-supervised machine learning approach to automatically annotate medical concepts in the narrative.

**Results:**

Evaluation of system adoption was performed by a mixed methods approach applying The Unified Theory of Acceptance and Use of Technology (UTAUT) as a theoretical lens. Most of the respondents demonstrated a high degree of system acceptance and expressed a positive attitude towards the system in general and intention to use the system in the future. Increased detection of patient allergies, and thus improved quality of practice and patient safety during surgery or ICU stays, was perceived as the most important advantage of the system.

**Conclusions:**

Our combined machine learning and rule-based approach benefits system performance, efficiency, and interpretability. The results demonstrate that the proposed CDSS increases detection of patient allergies, and that the system received high-level acceptance by the clinicians using it. Useful recommendations for further system improvements and implementation initiatives are reducing the quantity of alarms, expansion of the system to include more clinical concepts, closer EHR system integration, and more workstations available at point of care.

## Introduction

Undisclosed allergic patient reactions are a major risk when undertaking surgeries in hospitals [1]. Structured data elements containing critical information about patient allergies (e.g., anesthetics, drugs, contrast media, food, and environmental) in the electronic health record (EHR) may not be updated or complete, and may also be prone to inaccuracies increasing clinical risk [2, 3]. Furthermore, manual searching for and identification of clinical information in the patient “narrative” is hampered by a lack of robust search engines in todays’ EHR systems [4]. And, performing manual search for and identification of clinical information in the patient narrative is infeasible as it contains voluminous, unstructured, and complex data.

Although studies show clinical natural language processing (NLP) may successfully be used to harvest information and knowledge from EHRs to support clinical decision support systems (CDSS) at the point of care, such systems are generally still underutilized [5–9]. Clinical records display a range of different styles and grammatical structures, and achieving high performance requires expert domain knowledge for quality assurance of dictionary contents and extracted data [10, 11]. A more recent phenomenon is the implementation of machine learning-based NLP-driven systems. Automatic learning of complex clinical language structures have, however, also proven difficult [12]. Most of the systems of this type that have reached clinical utility in healthcare have used supervised machine learning which demand expert labeling of relatively huge amounts of data associated with high costs [13]. Compared to rule-based approaches which are still dominant, there are also challenges with interpretability, and the difficulty of correcting specific errors reported by end users (rule-based systems can easily modify rules to correct such errors) [9].

This backdrop was conducive to us developing the presently evaluated CDSS named Information System for Clinical Concept-based Search (ICCS). The CDSS, developed in a Norwegian hospital trust, incorporates a novel algorithm-based approach for text mining of the patient narrative for identifying and classifying patient allergies (to automatically flag/alarm when patient allergies are serious and requires further attention from physicians). Our approach is novel in that it employs unsupervised machine learning algorithms to analyze large corpora of clinical narratives to automatically generate a clinical language model comprising words and phrases of which meanings and relative meanings are also learnt [14]. As such, issues related to misspellings, compound words, and lexical variants are also greatly diminished. The CDSS furthermore combines unsupervised and supervised algorithms to semi-automate and simplify the building of clinical vocabulary, which to a large degree eliminates the annotation efforts (of the clinical narrative) necessary for the training of supervised algorithms [11, 14]. Finally, the system implements a precision layer of deterministic rules for fine-grained control. Besides allowing us to tag narrative text with similar accuracy as traditional expert systems, this layer also helps with interpretability and to correct errors reported by end users [14].

In a previous study [14], we performed empirical experiments on a real-world hospital derived clinical dataset to test the performance of the system in identifying and classifying patient allergies against a manually curated gold standard list of patient allergies. Based on the promising system performance (recall 92.6%, precision 88.8%, F-measure 90.7%), the CDSS was implemented, tested, and evaluated over a period of four months in a routine clinical setting in an anesthesia and intensive care unit (ICU) in the hospital trust. In the present study, we summarize the findings of the system evaluation specific to the system’s early implementation stage. Our objectives were to assess users’ perceptions towards the implemented CDSS, and to examine user interactions with the system and possible relationships between perceptions and use. Evaluation of system adoption and use was performed by applying the Unified Theory of Acceptance and Use of Technology (UTAUT) as a theoretical lens for the study [15–17]. UTAUT is a well-tested theoretical model, which has proven to be relevant for use in the healthcare context and for a diversity of technologies [16, 17].

## CDSSs and NLP in healthcare

CDSSs are systems that integrate and present patient-specific clinical information to healthcare professionals in a consistent manner, and they are designed to enhance patient care by providing context-relevant patient data and knowledge to aid in complex decision making [18, 19]. Attempts at implementing CDSSs in healthcare have been a story of mixed success [20–23]. However, such systems have been successfully implemented in different clinical settings and have been shown to reduce costs [20], improve quality of practice and patient safety [24]. Key issues for successful CDSS are correct and meaningful information, conceptual simplicity, integration into clinical workflow, and speed and ease of access [21, 25–29]. A principal aspect of such systems pertains to autonomy, which for system use and acceptance specifically refers to whether users are forced to accept the CDSS suggestion, whether they can easily ignore it, or whether it takes considerable effort to override the advice [24, 25, 28, 30, 31].

Rule-based NLP systems, often denoted expert systems, are the oldest and still most commonly used [9]. Machine learning-based systems for clinical NLP are a more recent phenomenon, with classification being a primary focus [32, 33]. Although many studies on supervised machine learning methods for clinical NLP exist (e.g., for extraction of clinical concepts), most of them are limited to experimental laboratory settings [34]. Examples of such systems being actively used by physicians for decision support in clinical settings are not so many (e.g., Lancet, TLINK, TIMEX3, and CTAKES being some notable exceptions), and relatively few studies focusing on empirical system evaluations have been published [8, 9, 35]. Finally, there is the trend of orchestrating different methods (e.g., unsupervised and supervised machine learning) for increased NLP performance and efficiency [12, 13, 32, 36–40].

### Conceptualizing UTAUT to system specific phenomenon and context

Black et al. [41] showed that there is a gap between the postulated and empirically demonstrated benefits of eHealth technologies. Since then, several technology acceptance frameworks originating from theoretical insights used in psychology, sociology, and information systems have been utilized to examine the individual acceptance and use of technology in healthcare settings [42]. While the technology acceptance model (TAM) is the most cited adoption model [16, 43–47], the UTAUT model integrates all constructs from previous models and is considered the most sensitive model for explaining variance in technology acceptance [16–18, 48].

**Fig. 1:**
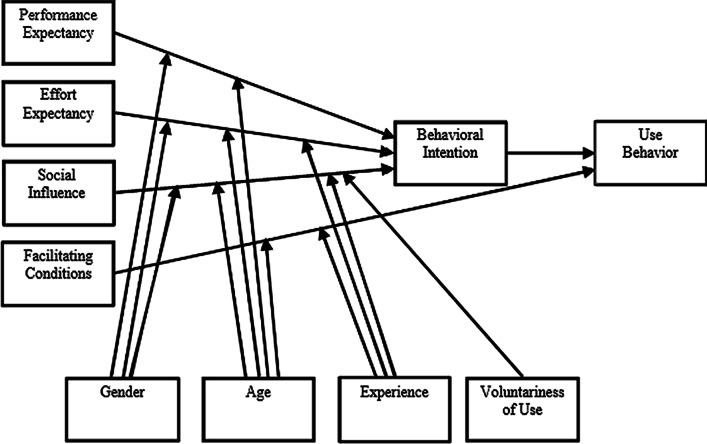
The original UTAUT; with its three determinants of behavioral intention to use a technology, two determinants of technology use, and contingencies that alter the effect of the determinants. Used with permission from MIS Quarterly [15]

The original UTAUT (see Fig. 1) identifies four principal constructs that directly or indirectly determine user acceptance and usage behavior: performance expectancy, effort expectancy, social influence, and facilitating conditions [15]. Performance expectancy is defined as the extent to which a user believes that using the system will increase job performance. Effort expectancy is explained as the degree of ease associated with using the system. Social influence is the extent to which a user perceives that important others believe the system should be used [15, 16]. These three constructs directly affect the behavioral intention to use the system. The last construct, facilitating conditions, is described as the degree to which a user believes that an organizational and technical infrastructure to support the use of the system exists [15, 16]. Facilitating conditions together with behavioral intention again act as direct determinants of system use, moderated by the four contingencies gender, age, experience, and voluntariness [15].

UTAUT’s application to the areas of NLP, artificial intelligence, and CDSS acceptance research is limited [18, 46, 49]. Leveraging a general theory such as UTAUT involves tailoring it to the specific system and organizational context to make it practically useful [16]. To our knowledge, neither the phenomenon of artificial intelligence NLP-driven CDSS, nor the specific context of an anesthesia and ICU department, has yet been observed through the UTAUT lens [17]. The research is therefore in part explorative in nature and employs an adaption of the original UTAUT with all its constructs and moderators (except voluntariness) as a theoretical lens to evaluate CDSS adoption and use. In UTAUT, voluntariness moderates only the effect of social influence on intention [15]. Given that system use is voluntary (not mandated) for all of the doctors and nurses in our context, the moderation by voluntariness proposed in UTAUT will not be present as a dependent contingency, and is therefore not considered.

## The case of ICCS: a CDSS for clinical concept-based searching

### Clinical setting: identification of patient allergies

Specialized doctors and nurses in a hospital, anesthetists and anesthetic nurses, are trained to administer and manage anesthesia during surgical procedures. While anesthetists and anesthetic nurses diagnose and treat medical problems that may arise during and immediately after surgery, they also evaluate the patient's medical condition prior to surgery. As part of the preoperative evaluation, it is vital to know as much as possible about patients’ medical history, lifestyle, and medications. Particularly important information includes reactions to previous anesthetics, drugs, and any other known allergies. For instance, adverse drug reactions (ADRs) perceived as a type of allergic reaction occur in 10–15% of hospitalized patients worldwide [50], and significant risks, costs, and increased hospital stays are associated with unknown ADRs [51]. While not taking part in preoperative or surgery procedures, intensive care unit (ICU) nurses and doctors share many of the same challenges as the anesthetists and anesthetic nurses.

Patient allergies are routinely and continuously documented in the patient narrative as they are identified by health professionals. However, as described previously, there are challenges pertaining to missing critical information as structured data as well as lack of robust search engines in today’s EHR systems. A single electronic health record in the general hospital we study may comprise anywhere from hundreds to thousands of documents. Thus, a thorough examination of patient narratives may be left inconsistent and incomplete by busy physicians who often have a multitude of competing work tasks. While traditional expert systems and machine-learning-based NLP-driven systems have shown promise in retrieving clinical data from the narrative, both approaches demand the heavy involvement of technical and clinical domain experts for the manual updating of controlled clinical vocabularies or the annotation of medical concepts in the narrative [10, 11, 52, 53].

### Developing and implementing the system

Taken together, the possibilities and limitations outlined in the previous sections guided our approach to developing and implementing a NLP-driven CDSS for identifying and classifying clinical concepts such as patient allergies. Clinical concept searching represents an automated information retrieval method adopted to search unstructured text (i.e., the narrative) for information that is conceptually similar to that provided in a search query [54]. Concept-based search systems differ from keyword search systems in that they try to determine what a user means. Concept-based search systems incorporating a high degree of precision, most of the time returns hits on documents that are ‘about’ the subject/theme that is being explored, even if the words in the document do not precisely match the query [54]. Although ICCS has been developed for universal clinical concept search, only the clinical concept of allergy was included during the four months of testing the system. Including synonym words and phrases, the clinical concept of allergy effectively includes almost 7000 relevant words and phrases.

While developing the system, we specifically aimed for reducing dependence on clinical resources. In brevity, by using unsupervised machine learning algorithms, large corpora of clinical narratives are text mined and analyzed to automatically build a clinical language model containing words and phrases of which meanings and relative meanings are also learnt. By exploiting the weighted associative power (i.e., inferring the semantics of the words based on their distribution) of related clinical terms and phrases, we aimed to achieve somewhat the same effect as we would by using a custom-built controlled vocabulary of allergy-related words, but with much less work (i.e., not involving human intervention) [11, 14]. ICCS also implements rule-based algorithms, and a semi-automatic annotation scheme for efficient and interactive machine learning, which to a great degree eliminates the substantial annotation efforts (of the clinical narrative) commonly associated with the training of supervised algorithms [14]. At runtime, the system combines unsupervised and supervised machine learning algorithms to guide the clinical language model towards the concept of allergy, and rule-based algorithms to precisely filter the patient narrative and to present physicians with concept relevant information.

Because system speed is essential for busy clinicians [55], much time was spent on optimizing the time taken to use the CDSS. For example, the results of the patient data retrieval and analysis should be presented to the clinicians effectively in seconds to support real-time decisions [56]. Finally, retrieving and analyzing a patient health record containing about 400 documents took about 10–15 s. A doctor or a nurse manually reading and searching through the same amount of documents for specific information would generally spend hours completing the task.

**Fig. 2:**
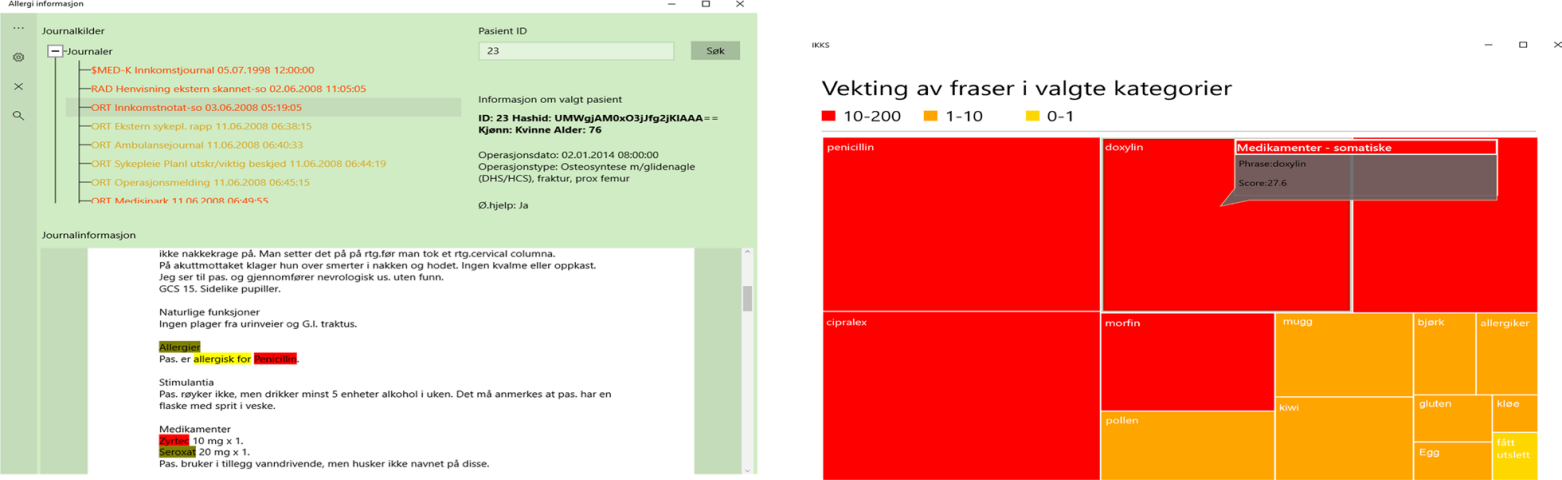
The main CDSS user interfaces. The picture on the left shows a screenshot of the main user interface of ICCS. Patient documents containing patient allergy information are filtered and presented, and allergy concept-related words or phrases are highlighted in the text. To the right is shown a screenshot of the module for visualizing patient allergies as structured and classified data. Color-coding is used to display the severity of allergies, and allergies are also classified according to allergen type

Furthermore, in designing the user interface, emphasis was put on user feedback concerning ease of use and simplicity [55], with main functionality being limited to: login and logout; incremental drop-down list patient search; a tree structure for traversing (concept relevant) narrative documents, laboratory tests, and critical information; a document module where concept relevant phrases are contextually marked with color-coding according to classification (e.g., allergy severity); a critical information module that flags concept-related critical information (e.g., drug allergies) with color-coding; a laboratory tests module that display all past concept-related laboratory results and flags those results that fall outside of the reference range (e.g., abnormal allergy-related tests); and a module for visualizing and classifying the concept relevant data (e.g., allergy types and severity) as nested rectangles with different colors, sizes, and scores in a Treemap structure. Applied to the clinical concept of allergy for example, nested rectangles reflect allergy type, while colors, area sizes, and scores indicate severity. A larger area size/red color reflects severe allergy, while a smaller area size/amber or green colors indicate a less severe allergy. See Fig. 2 for screenshots of two of the main CDSS user interfaces (the document module and the module for visualizing and classifying the concept relevant data).

### System architecture and dataset

The CDSS architecture includes steps for EHR data extraction and conversion, natural language pre-processing, building of probabilistic machine learning models, and finally algorithmic processing including 35 deterministic rules used to highlight concept-related information presented by ICCS. The system client is developed in C# and was tested on PCs and tablets during the evaluation period. Further details on the system architecture are covered elsewhere [14], and will not be presented again in this paper that has more of an empirical focus.

ICCS uses data obtained from the hospital trust’s enterprise-wide integrated EHR system. The narrative part of the EHR system contains a copy of all the clinical documents for hospitalized patients admitted to either somatic, psychiatric or radiology departments. Since the system’s inception in 1992, 62,338,499 clinical documents (at the time of writing), have been stored in the system across 3756 different document types. Common document types include (but are not restricted to) hospital admission and discharge summaries, progress notes, outpatient clinical notes, medication prescription records, radiology reports, laboratory data reports, surgery notes, anesthesia and intensive care journals, physician referrals, and a range of different specialized forms containing structured data and/or unstructured information.

## Method

### Study design and sample

The user groups in this study are the doctors and nurses in the Anesthesia and ICU department in a Norwegian hospital trust, whom during four months (between May 1st 2017 and August 31st 2017) used ICCS for identifying and classifying patient allergies. Necessary approvals from the relevant national committees on ethics and data protection were obtained.

Several recent healthcare IT reviews recommend methodological pluralism to study complex healthcare IT to make it more relevant and impactful [57–59]. We used a convergent mixed methods study design employing qualitative and quantitative methods in parallel [57, 60, 61] to evaluate system adoption. Survey research based on small sample sizes (as in this study), may be augmented with other measures to help establish the validity of the results [62]. See Table 1 for a detailed overview of how we used each of the methods, the rationale for using them, their requirements, and how they complement each other. We used four forms of qualitative research—ethnographic observations, user logbooks, review of documents, and interviews. The first three forms were conducted to obtain an understanding of doctors and nurses everyday work situation, work practices, and perspective (emic view) [57, 63, 64].

**Table 1:** Overview of methods

Method	Rationale and focus (R)	Required input (I)	Scope	Timeline
Questionnaire	Quantitative data Assess users’ perceptions towards the implemented CDSS (R1) To investigate user interactions with the system and possible relationships between perceptions and use (R2)	The UTAUT model (I1) The literature review of factors related to implementing and using CDSSs (I2)	52 doctors and nurses (8 doctors and 44 nurses) 52 questionnaires distributed and answered (100% response rate) 32 construct derived questions (1 open-ended)	After three months of system use
System usage data	Quantitative data To assess the actual use of the system (R3) and to qualitatively analyze system usage behavior (R4) Also to help identify the most frequent users of the system/interview subjects	Data from the system logs	All of the 81 users Central functionalities: User login time (R3) and frequency (R3, R4) User data/documents retrieval requests (R4) Client workstation location (R4)	Performed for the testing and evaluation period of four months
Semi-structured interviews	Qualitative data Same as R1 and R2, with the main emphasis being on gathering in-depth information	Same as I1 and I2, to inform construction of the interview guide Analysis of the system logs to identify the most frequent users	14 individual interviews: 6 doctors 6 nurses A nurse and a doctor holding leadership positions Total, 609 min Average 43.5 min Shortest/longest 30/70 min 87 construct relevant questions (derived from 6 and 7)	After two months of system use
Ethnographic observations	Qualitative data Same as R1 and R2 Facilitate an understanding of doctors and nurses everyday work situation, work practices, and perspective (R5) Understanding the daily use of patient records in the EHR system (R6) First-hand observation of doctors and nurses using the CDSS Changes in work routines due to CDSS Inform the questionnaire and interview guide (R7)	Observation of doctors and nurses in the Anesthesia and ICU department and in the anesthesiology out-patients’ clinic	119 h 28 h of user training 59 h in the Anesthesia and ICU department 32 h in the anesthesiology out-patients’ clinic	Performed during the four months system testing and evaluation period
User logbooks	Qualitative data Same as R1, R2, and R5	Data from the user logbooks available at the client workstation locations	17 logbook entries	Performed during the four months system testing and evaluation period
Document review	Qualitative data Same as R6 and R7	Data from the hospital trust’s enterprise EHR system	22,000 EHR documents	Performed during the system design and implementation phase

### Interview guide and questionnaire development

The structure and the formulation of questions both in the interview guide and the questionnaire were informed by the UTAUT model and a literature review of salient factors, including health professionals’ acceptance, related to implementing and using CDSSs. The literature review factors were classified under the different constructs of the UTAUT model: performance expectancy, effort expectancy, social influence, and facilitating conditions. All questions in the questionnaire, except those aimed at collecting sociodemographic information, used five-point Likert scales with responses ranging from ‘‘Strongly disagree’’ (1) to “Strongly agree” (5). The questionnaire was supplemented with an open-ended question to allow free expression of ideas or perceptions. The draft questionnaire and the interview guide were separately reviewed and piloted by a doctor and nurse expert user to establish content validity. Based on the received feedback, the questionnaire and the interview guide were updated.

### Data collection, analysis, and measurement

During the data analysis, data from the interviews, document review, and the ethnographic observations helped us to better understand and interpret the feedback given by the doctors and nurses in the survey, and vice versa [59]. Taken together, our concurrent qualitative and quantitative analyses served as complementary approaches [60] for developing a more complete picture of the users’ perceptions towards the implemented system.

The interviews were transcribed verbatim, and the analysis of the interview data was manually undertaken following the four stages recommended for qualitative data analysis [63, 65]: theme identification (according to constructs in the UTAUT model and the classified literature study themes); coding data to themes; data displays and interpretation of themes and displays; and drawing conclusions. Two researchers with clinical backgrounds independently coded all interviews into relevant themes that were later discussed to achieve consensus. NVivo was used to support the analysis of the qualitative data; that is to organize, code, and translate the data into the results. Finally, interview data analysis was supplemented by written user feedback recorded in logbooks that were distributed to all computer sites and available to all users.

After three months of system use, a survey was administered to a sample of 8 doctors and 44 nurses drawn from a random selection of 20 doctors and 100 nurses in the Anesthesia and ICU department who had received training in how to use the system. The responses to the survey were divided into two groups for analysis based on frequency of system use and clinical setting: the frequent users who spent working hours in the anesthesiology out-patients’ clinic (group A), and the participants only working in surgery or in the intensive care unit who generally used the system less (group B). To calculate mean composite construct scores (provided in Table 3), responses to each construct’s items in the questionnaire were summarized and divided by the number of items. While cases were removed from analysis when more than one scale item was left unanswered, the mean score for the other completed items was allowed when only one construct item was missing. The sample population and the data in the log files were analyzed and described using descriptive and inferential statistics in SPSS Statistics 23 as appropriate. Results were summarized in tabulated and graphical descriptions. Cronbach’s alpha was employed to evaluate the reliability of the construct measurements. Chi-square, Fisher exact test, Mann–Whitney, Kruskal–Wallis, and Spearman correlation tests were performed as appropriate to detect possible relationships between observed and latent variables respectively. All statistics were conducted using a two-sided test and a significance level of 0.01 (strongly significant relationship) and 0.05 (significant relationship). Due to the exploratory nature of our analysis, post hoc procedures such as Bonferroni corrections were not applied on non-parametric testing results to minimize Type II error inflation [66].

## Results

### Respondents’ characteristics

The sample group included 8 (15%) doctors and 44 (85%) nurses out of a total study population of about 250 doctors and nurses in the Anesthesia and ICU department. The response rate was 100% among the sample group. 44% of the respondents were male, and 56% were female. Most of the respondents (40%) were in the age group from 40 to 50 years, and 48% of the respondents had more than 20 years of experience working in the hospital. Characteristics of the respondents’ age and experience are shown in Table 2. All respondents had received training in the use of ICCS, and had used the system at least once.

**Table 2:** Respondents’ characteristics

Age groups		Years of experience as doctor or nurse	
30–40	23%	5–10	13%
40–50	40%	10–15	12%
50–60	23%	15–20	27%
60–70	14%	> 20	48%

### Construct measures

Table 3 summarizes the construct measures for the total number of users (N = 52), and the two subgroups.

**Table 3:** Cronbach’s alpha, minimum, maximum, median, mean and standard deviation of the constructs

Construct	N	Cronbach’s α	Min	Median	Mean ± SD	Max
*All*						
Performance expectancy	52	0.7	1	5	4.58 ± 0.37	5
Effort expectancy	52	0.72	2	5	4.76 ± 0.38	5
Social influence	52	0.77	1	5	4.13 ± 0.70	5
Facilitating conditions	52	0.63	1	4	3.81 ± 0.59	5
Behavioral intention to use system	52	0.79	1	5	4.78 ± 0.46	5
*Group A: Users working in the anesthesiology out-patients’ clinic*						
Performance expectancy	11	0.76	2	5	4.70 ± 0.28	5
Effort expectancy	11	0.67	4	5	4.93 ± 0.13	5
Social influence	11	0.84	3	5	4.45 ± 0.70	5
Facilitating conditions	11	0.41	1	5	4.30 ± 0.41	5
Behavioral intention to use system	11	0.79	5	5	5.00 ± 0.00	5
*Group B: users working in surgery or the intensive care unit*						
Performance expectancy	41	0.69	1	5	4.55 ± 0.39	5
Effort expectancy	41	0.75	2	5	4.72 ± 0.41	5
Social influence	41	0.72	1	4	4.10 ± 0.68	5
Facilitating conditions	41	0.59	1	4	3.70 ± 0.57	5
Behavioral intention to use system	41	0.78	1	5	4.72 ± 0.50	5

Almost all of the respondents (98%) had a positive attitude towards the system in general, and intended to use the system in the next months provided its availability. Judging by the constructs mean scores, group A was generally more favorably inclined to the system than group B. This is especially true for the construct facilitating conditions when observing the two groups’ mean scores (4.30 ± 0.41 vs. 3.70 ± 0.57).

Mean perceived usefulness or performance expectancy was 4.58 ± 0.37. All of the respondents experienced the system as useful. 96.2% of the respondents reported that the system provided correct and meaningful information. It should be noted, however, that while not perceived as a disadvantage by most of the respondents (59.6%), noise, or false identification of allergies by the CDSS, was perceived as a disadvantage by 11.5%. 96.2% of the respondents recognized system speed as a positive factor. More specifically, 90.4% reported increased productivity by using the CDSS, caused by having to use less time on manually searching the patient narrative for allergies. However, even more important than the productivity gains reported by the users, was the system’s contribution to increasing treatment quality and patient safety (reported by 98.1% of the users) caused by the increased number of patient allergies detected.

Mean effort expectancy score or perceived ease of use was 4.76 ± 0.38. Generally, the doctors and nurses were favorably disposed towards the system’s ease of use. All of them responded with a positive sentiment when asked about the system’s ease of use, 98.1% of the users experienced the interaction with the system as intuitive and clear, and 92% responded that the system was easy to learn.

The system achieved a mean score of 4.13 ± 0.70 for the social influence construct. Differences in opinion especially existed about whether or not the hospital had supported use of the system. While 45.2% agreed or somewhat agreed positively, 55.8% responded indifferently. Contrasting this, however, 86.6% of the users experienced leadership as helpful or positive towards usage of the system (with only 2% being negative). Of all the constructs, facilitating conditions reflected the most negative mean score at 3.81 ± 0.59. 26.9% of the users replied that they did not have enough resources to use the system, 28.9% found the system to be unavailable when needed during their workflow, and 40.4% reported that the system was not available all of the places where it was needed in the hospital.

As for the last construct, behavioral intention to use the system, a mean score of 4.78 ± 0.46 was achieved. Generally, the results of the three items that made up the construct corresponded well with only small variations. Cronbach’s alpha tests showed the questionnaire to reach acceptable reliability, α = 0.82 (total score). An examination of the loading items suggested that they adequately represented the conceptual underpinnings of the constructs for the whole sample group in our context [67, 68].

### Actual system use

At the end of the four months test period, the CDSS contained a copy of 5,553,953 clinical documents belonging to 31,841 emergency or elective patients admitted (including future planned admissions) to the Anesthesia and ICU department in the hospital. These were patients admitted or planned for either anesthesia screening, surgery, or intensive care. The system log files indicated that 81 users had used the system 728 times during the test period to access 2740 patient documents. 19 of the users had used the system more than 10 times during the test period, while 10 of the users had used it more than 20 times. The user who had logged into the system most frequently during the test period had used it 80 times to access 34 patients’ 177 different documents, while the user with the highest number of accessed documents (266), had logged into the system 35 times accessing 30 different patients. With a few exceptions, the doctors and nurses in group A used the CDSS more frequently than group Main et al. [22], referring to Ohmann et al. [69], highlighted that CDSS satisfaction is a complex interplay between both system-dependent and system-independent factors. This is shown in the characteristics of actual system use are shown in Table 4. System robustness is important, because lack of it is one of the most important reasons in the literature for not using a CDSS [21, 25]. Except during monthly routine emergency generator tests in the hospital, no downtime of the system was reported.

**Table 4:** Characteristics of actual system use

	Users	Total	Percentage	Mean	Median
*User logins*					
All	81	728	100	9	4
Doctors	21	344	47	16	6
Nurses	60	386	53	6	4
Male	35	347	47	10	4
Female	46	383	53	8	4
Group A	11	394	54	33	26
Group B	70	336	46	5	3
*Documents accessed*					
All	81	2293	100	33	13
Doctors	21	1038	45	61	17
Nurses	60	1255	55	24	12
Male	35	1040	45	35	14
Female	46	1253	55	31	13
Group A	11	1461	64	122	147
Group B	70	832	36	14	11

### Correlations between the constructs

Table 5 shows the correlations between the constructs.

**Table 5:** Correlations between the constructs (N = 52)

	BI	Use
*Performance expectancy*		
Spearman correlation	0.466**	0.590**
Sig. (2-tailed)	< 0.001	< 0.001
*Effort expectancy*		
Spearman correlation	0.544**	0.528**
Sig. (2-tailed)	< 0.001	< 0.001
*Social influence*		
Spearman correlation	0.361**	0.223
Sig. (2-tailed)	0.009	0.112
*Facilitating conditions*		
Spearman correlation	0.222	0.240
Sig. (2-tailed)	0.114	0.087
*Behavioral intention to use the system*		
Spearman correlation	NA	0.487**
Sig. (2-tailed)		< 0.001

**Correlation is significant at the 0.01 level (2-tailed)

BI: Behavioral intention to use the system, Use: system use

Statistically significant correlations existed between intention to continue using the system and performance expectancy (perceived usefulness) (*p* < 0.01), effort expectancy (perceived ease of use) (*p* < 0.01), social influence (*p* < 0.05), and system use (*p* < 0.01). The correlation with the construct facilitating conditions was not significant. System use was moreover significantly correlated with performance expectancy (perceived usefulness) (*p* < 0.01), and effort expectancy (perceived ease of use) (*p* < 0.01). The correlations with the constructs social influence and facilitating conditions were not significant. The scores in the constructs were not related to gender, age, or experience. However, Mann–Whitney tests indicated that workplace (group A versus group B) was significantly (medium to large effect) related to the scores in the construct facilitating conditions (U = 81.5, Z =  − 3.23, *p* < 0.05, r =  − 0.45), with (large effect) scores in the construct system use (U = 28, Z = -4.65, *p* < 0.01, r =  − 0.64), as well as somewhat (small effect) with scores in the construct behavioral intention to use the system (U = 154, Z =  − 2.1, *p* < 0.05, r =  − 0.29). Moreover, profession was significantly related (small to medium effect) to the scores in the construct facilitating conditions (U = 80.0, Z =  − 2.09, *p* < 0.05, r =  − 0.29).

### Interviews and observations: summary of findings

While system speed and ease of use were highlighted, improved quality of practice and patient safety because of increased patient allergy detection were pointed to as the most significant system benefits. Two of the respondents screening patients in the anesthesiology out-patients’ clinic confessed that they initially had used the system only on patients that they knew had confirmed allergies. However, after having experienced that the system detected additional severe patient allergies that they were not aware of, they started to use the system on all their patients.

As many patient encounters are unplanned or urgent, allergy relevant information may not be readily available, or there may not be time for the doctors and nurses to comprehensively examine the patient’s health record for information on patient allergies. The system’s fast response meant that when competing work tasks compromised a thorough examination of the patient narrative, they could now leave the exercise to the system, and have answers in seconds. It was also pointed out that when interacting with comatose, elderly or sick patients, and refugees/patients of foreign origin, the system could help to confirm patient allergies.

While the system was easily available at point of care for all of the doctors and nurses in group A, system availability for group B was limited with the system being available at only three workstations and two tablets. In the intensive care unit, doctors, and especially nurses, found it challenging to use the system due to lack of workstations with the system installed. One of the offices in the unit where the system was available was regularly occupied by doctors, making it difficult for nurses or other doctors to get space and time to use the system. The informants generally perceived system use and adoption as being contingent upon system diffusion and integration into clinical workflow. Installation at all the available workstations in the intensive care unit and the surgery area, including workstations in the operation theaters, was emphasized as a pre-requisite to this end. Because of the system’s potential positive effects on clinical workflow, quality improvement, and patient safety in general, they further ideally envisioned hospital-wide system access at all workstations. Securing access to the system in the emergency departments should also be a priority, as the admission notes documented there serve as an important source of information for health professionals throughout patients’ entire hospital stays. A related point here is also that many of the informants emphasized integration of ICCS into the hospital’s enterprise EHR system, for ease of system access and stronger integration into existing clinical documentation and workflow.

For the majority of the informants, alarm fatigue caused by repetitive, peripheral, or erroneous allergy information presented by the system was not an issue. However, one of the nurses expressed dissatisfaction with ineffective information causing alarm fatigue, while another nurse cautioned against it. They generally did not like ineffective alarms when they were intensively occupied during consultations and had little time to use the system. None of the respondents, however, expressed such emotions during the later interview rounds, even when directly confronted. Because the system design allowed them to easily verify the data transformations and the output taking place through the open processing pipeline, they had come to trust the system through their own verifications.

### Implications for further research and practice

While the results of the study are contributing to healthcare IT, NLP, CDSS, and UTAUT research, they are also helpful for formulating recommendations towards system improvements and further implementation initiatives. Primarily, they clarify that there is a need for greater system diffusion or saturation, including closer integration into the existing EHR system [21, 23, 25, 26]. While considerable efforts were spent on optimizing the system for precise concept-based searching, the survey results indicated that noise, or false identification of allergies by the CDSS, was perceived as a disadvantage by a small number of the users. Prevention of alert fatigue should be an important aspect of the design of CDSSs, as several studies report CDSS alerts are often ignored with high override rates ranging between 49 and 96% [70]. Fundamental in this respect, is that the trigger level for CDSS alerts must be set to the appropriate sensitivity [71]. Pons et al. [8], referring to Percha et al. [72], report on a radiology system with extremely high performance (99% correctly classified cases) explained by the consistent use of standardized terminology in describing breast tissue composition. However, allergies are heterogeneous concerning both their underlying pathophysiology and their clinical manifestations (ranging from mild rashes to life-threatening anaphylaxis) [26]. Even though the system is quite precise at detecting and classifying the clinical concept of allergy with a measured recall score of 92.6% and precision at 88.8% [14], we believe the results reflect that there is room for further improvements especially related to filtering out peripheral and/or repetitive patient allergy information to reduce the quantity of alarms. System tuning to increase performance may similarly, to varying degrees, turn out be necessary in future expansions of the system to include more clinical concepts.

From the outset of developing the CDSS, our philosophy was to deliver only “lightweight” clinical decision support. While on one side the results indicated that most of the respondents did not perceive that the system tried to replace them as clinicians, on the other side most of them also responded that the system helped them to make correct decisions. Determining a balance between the sensitivity and the specificity of the trigger level for CDSS alerts is crucial. Limiting the quantity of the reminders could be considered to improve the specificity at the cost of the sensitivity [73]. As such, further filtering of patient allergy information, as mentioned in the previous paragraph, may also introduce a risk. The analysis of the interview data suggested that, although warned about system inadequacies, some of the users may already have started to rely too much on the system for allergy detection [74, 75]. While emergency situations may defend such use, we generally believe that ICCS use should not substitute EHR system narrative reading and searching processes as long as the system’s recall of allergies cannot be guaranteed.

While traditional expert systems may experience performance issues if words or phrases that appear in the narrative text are not accounted for in dictionary sources (e.g., due to misspellings, compound words and lexical variants) [76], machine learning-based clinical NLP systems have been denounced for depending heavily on domain expert-driven annotations. Systems that use NLP-techniques as part of their repertoire need to address such shortcomings for acceptance in healthcare institutions and among its clinical users. More specifically, machine learning NLP-driven CDSSs have to deliver in terms of both performance, efficiency, and interpretability [8, 9]. By implementing high performance unsupervised learning of word embeddings, our method is able to cover commonly misspelled words, abbreviations, and acronyms [14, 42].

The CDSS we have developed leverages semi-supervised learning for simplified, interactive, and accelerated user-based (e.g., clinical domain experts) clinical concept building and automatic annotation [14]. This approach is more efficient than traditional machine learning approaches, because the unsupervised method to build clinical vocabulary needs limited support from technical and clinical domain experts [14]. Once implemented with clinical concepts to search for, the system is to a large degree self-learning and self-maintaining, because the language model has the potential capacity of being automatically updated with new knowledge as the clinical language changes and evolves. Thus, the longstanding problem of dependency on domain experts for developing and maintaining specialized clinical dictionaries used for clinical concept tagging is to a large degree eliminated. Further, augmenting the machine learning results with a layer of deterministic rules enables us to leverage the benefits of a traditional expert system, such as fine-grained control of text tagging, easy modifications of rules to correct specific user reported errors, and adding new vocabulary (such as a new drug) not yet intercepted by the unsupervised algorithm.

Finally, it is imperative for healthcare that such systems like physicians should not only support clinical decisions; they should like human beings also be able to explain their decision making for trust building among its clinical users [9]. Governments use large amounts of their GDPs to support their healthcare systems [77], and people generally have a high degree of trust in healthcare. As witnessed by recent events [78], healthcare has small tolerance for errors in artificial intelligence-driven IT-systems aiming at delivering clinical decision support. Such systems should like clinicians be able to explain their results, and optimally also be able to tell us when they are unsure. Indeed, trusting a system in most cases is a prerequisite for system acceptance. Based on the findings of the study, we believe Shibl et al.’s [16] added construct *trust in the knowledge base* to the original UTAUT might be considered for inclusion in future similar studies. Alternatively, as we did in the interview guide, the UTAUT construct “Anxiety” may be included [15], as the analysis of the interview data shows it is able to cover some of the same ground. ICCS was designed to show its interpretations not only as aggregated conclusive data. Unlike a “black box” type approach, its open processing pipeline gradually refines the clinical data. This allows the output and its underlying interpretations to be traced back to the original clinical raw data quite transparently. Including the mentioned constructs in evaluation studies of artificial intelligence-driven systems, allows system developers to get valuable user feedback on whether they have succeeded with incorporating enough transparency, or interpretability of results, into their systems to support trust building.

ICCS includes features that extract concept-related data from different parts of the patient’s EHR (e.g., the narrative and laboratory results) and is capable of displaying them in a summarized dashboard-like format. It is also able to filter, classify, and flag/alert concept relevant data and information (e.g., allergies and abnormal laboratory results). The system has therefore met the requirement of a tier 2 CDSS [79, 80]. Stronger, or more advanced, tier 3 clinical decision support (i.e., used for more cogent patient recommendations or diagnosis purposes) entails analyzing unstructured and structured data in the patient EHRs at aggregated levels [79, 80]. However, a necessary precursor to the synthesis of data at aggregated levels for precision medicine, is the more basic NLP-capability to search for and classify complex information precisely and exhaustively at human cognitive performance (or surpassing) levels as demonstrated here. While the results of the study reflect some limitations to our machine learning approach in emulating human natural language cognition, we still believe they are encouraging to the extent that they call for further exploration in a tier 3 direction.

### Limitations

The limitations of this work should be considered when interpreting our findings. The most important limitation concerns the sample. The sample size of the survey study is small, especially for Group A, which constrains statistical inference power. Second, it has to be observed that results of our questionnaire were self-reported measures of use and influence, and that users’ evaluation of their own behavior may contain bias [73]. The results were restricted to the perceptions of a sample of current CDSS users and did not include the valid input of the whole population of doctors and nurses or all of those who used the system. A somewhat large percentage (78.9%) of the users of the system only worked in surgery or in the intensive care unit, and as reflected by the results they generally were not frequent system users.

Although the limited group of respondents’ perceptions do not necessarily represent the opinions of all the doctors and nurses in the department, the survey results represent real-world perspectives and constitute valuable input for recommendations towards system improvements and further implementation initiatives [73]. To this end, we also believe the randomized (i.e., the survey participants) and mixed method design of the study mitigate some of the described weaknesses and support validation of the results. For example, the respondents’ high intention to use the system was reflected in correspondingly high actual system use, and the system benefits reported by the informants helped to confirm the perceived usefulness by the respondents. We also observed that the interview and observational data enabled explanations to several of the more “obscure” statistical findings and allowed for a richer and deeper understanding of the case. For example, while there was quantitative evidence of no system downtime, a significant number of the respondents’ perceived lack of availability of the system when or where needed. The information provided by the informants shed light on this apparent divergence in the findings and led to recognition of a problem in how the system was implemented, rather than a problem with the system itself. Nevertheless, the results should be interpreted with caution, as they are specific to the early implementation stage of the system.

As argued in an earlier paper [14], we believe the system with some adjustments (i.e., to the EHR data extraction mechanism and deterministic rules for precise text filtering), should be flexible enough to be transferable to other departments, hospitals (or healthcare organizations using EHR systems in general), languages, and countries. However, because data in both the present and earlier study included only the clinical concept of allergy and was collected from a single hospital and in the context of a specific EHR system implementation, the generalizability of our findings is limited. It is possible that our results would be different in other implementation contexts. Hence, future research should test the system using other clinical concepts, and in other departments and healthcare organizations.

Hospitals are often run-on tight budgets with a clear focus on and commitment to their patients. The doctors and nurses working in anesthesia and ICU departments, however, are perhaps particularly busy in their work given the sense of urgency, unexpected happenings, and frequent rescheduling of work tasks. Thus, the researchers had to do a great deal of follow-up to collect the necessary data for the study. Enrolling greater portions of a hospital department’s population of health professionals into research studies without somehow disturbing clinical practice is difficult. In this case, the system’s now proven advantages were hopefully able to make up for some of the time the doctors and nurses most willingly invested in having to learn the system and using it, filling out the questionnaires, and being interviewed.

## Conclusion

We have presented our results from the evaluation of a NLP-driven CDSS developed and implemented in a Norwegian hospital trust. The system employs unsupervised and supervised machine learning algorithms combined with rule-based algorithms for clinical concept-based searching to identify and classify allergies of concern for anesthesia and intensive care. Evaluation of system adoption and use was performed by a mixed methods approach applying UTAUT as a theoretical lens. Most of the respondents demonstrated a high degree of system acceptance and expressed a positive attitude towards the system in general and intention to use the system in the future. Increased detection of patient allergies, and thus improved quality of practice and patient safety during surgery or intensive care unit stays, was perceived as the most important advantage of the system. In addition, system speed and ease of use were highlighted as positive factors. Results pertaining to the construct facilitating conditions gave mixed results, suggesting that there is a need for greater system diffusion, including closer integration into the existing EHR system. Improvements to the trigger level sensitivity for CDSS alerts is also a future point of interest. While the results of the study are contributing to healthcare IT, NLP, CDSS, and UTAUT research, they also provide useful recommendations for further system improvements and implementation initiatives. Finally, we would like to add that following the positive evaluation results, ICCS is now in regular use in the Anesthesia and ICU department in the hospital trust. Plans for expanding functionality to search for multiple clinical concepts, and also for implementing the system in other departments in the hospital, are currently being contemplated.

## Data Availability

There are ethical restrictions on sharing the study’s data (the data contains potentially sensitive information). In accordance with restrictions imposed by the Regional Committees for Medical and Health Research in Norway (approval no. 2016/329), data must be stored on a secure server at Sørlandet Hospital Trust. The contents of the ethics committee's approval resolution as well as the wording of participants' written consent do not render open public data access possible. Access to the study's data may be requested by contacting Geir Thore Berge (first author) at Sørlandet Hospital Trust (geir.thore.berge@sshf.no).
